# Comprehensive Adsorption Studies of Doxycycline and Ciprofloxacin Antibiotics by Biochars Prepared at Different Temperatures

**DOI:** 10.3389/fchem.2018.00080

**Published:** 2018-03-27

**Authors:** Zhi-wei Zeng, Xiao-fei Tan, Yun-guo Liu, Si-rong Tian, Guang-ming Zeng, Lu-hua Jiang, Shao-bo Liu, Jiang Li, Ni Liu, Zhi-hong Yin

**Affiliations:** ^1^College of Environmental Science and Engineering, Hunan University, Changsha, China; ^2^Key Laboratory of Environmental Biology and Pollution Control, Hunan University, Ministry of Education, Changsha, China; ^3^College of Architecture and Urban Planning, Hunan City University, Yiyang, China; ^4^School of Metallurgy and Environment, Central South University, Changsha, China; ^5^College of Environmental Science and Engineering Research, Central South University of Forestry and Technology, Changsha, China

**Keywords:** biochar, biomass, pyrolytic temperatures, antibiotics, adsorption, mechanisms

## Abstract

This paper comparatively investigated the removal efficiency and mechanisms of rice straw biochars prepared under three pyrolytic temperatures for two kinds of tetracycline and quinolone antibiotics (doxycycline and ciprofloxacin). The influencing factors of antibiotic adsorption (including biochar dosage, pH, background electrolytes, humic acid, initial antibiotics concentration, contact time, and temperature) were comprehensively studied. The results suggest that biochars produced at high-temperature [i.e., 700°C (BC700)], have higher adsorption capacity for the two antibiotics than low-temperature (i.e., 300–500°C) biochars (BC300 and BC500). Higher surface area gives rise to greater volume of micropores and mesopores, and higher graphitic surfaces of the BC700 contributed to its higher functionality. The maximum adsorption capacity was found to be in the following order: DOX > CIP. The π-π EDA interaction and hydrogen bonding might be the predominant adsorption mechanisms. Findings in this study highlight the important roles of high-temperature biochars in controlling the contamination of tetracycline and quinolone antibiotics in the environment.

## Introduction

Increasing public concern has been drawn to the non-regulated trace organic emerging contaminants (ECs), including pharmaceuticals, halogenated flame retardants, illicit drugs, and personal care products (Daughton and Ternes, 1999; Boxall et al., 2012; Marti et al., 2014; Petrie et al., 2015). As one type of pharmaceuticals, antibiotics have been widely used in the control of infectious diseases in humans and animals (Hong et al., 2013). China has a high antibiotics production capacity, with annual production of nearly 210,000 tons (Luo et al., 2011). However, these “applied” antibiotics might be released continually into soils, surface waters, and ground waters due to incomplete metabolism (in animals) and discharges from drug manufacturers (Carabineiro et al., 2011; El-Shafey et al., 2012). Discharged antibiotics may lead to the development of antibiotic-resistant microorganisms (Sidrach-Cardona and Bécares, 2013; Soori et al., 2016), adverse impacts on human health through endocrine disruption, increase of antibiotic resistance genes (Huang et al., 2015; Hou et al., 2016), and the potentially toxic effects of their unwanted/unknown byproducts (Le-Minh et al., 2010; Zhou et al., 2013). Therefore, it is imperative to tackle the antibiotics polluted water before more discharges are made into the aquatic environment.

Biochar is a carbon-rich material obtained by pyrolysing biomass waste with little or no available oxygen (Sohi, 2012). The specific properties of biochar (including a porous structure, surface aromaticity, and high functionality) make it possible to be used as an excellent biomass-derived carbonaceous adsorbent for organic contaminants (Tan et al., 2016; Peiris et al., 2017). It is reported that pyrolytic temperature has significant effect on the properties of the biochar and is likely to be a major factor contributing to the adsorption properties of the biochar (Tan et al., 2015). Chen et al. (2012a) reported that the pyrolytic temperature have significant effect on the adsorption ability of the resultant biochar because the temperature affected the degree of carbonization of the biochar. There was a sharply enhanced adsorption efficiency of Naphthalene at higher formation temperature, for example, due to the completely carbonized organic matter of the biomass, the greatly increased surface area, and the more developed nanopores. Xie et al. (2014) produced pine wood biochars at different thermochemical conditions and applied them to sulfonamides adsorption. The results suggested that adsorption was stronger to biochar pyrolyzed at 500°C (C500) rather than 400°C, which was attributed to the higher degree of graphitization of the C500 surface and the enhanced π-π electron-donor-acceptor (π-π EDA) interaction. However, certain studies have reported the opposite pattern, i.e., that the adsorption ability of low-temperature biochar was higher than that of high-temperature biochar. Yao et al. (2012) found that the lower temperature biochar exhibited higher adsorption of sulfamethoxazole, as biochars produced at lower temperatures might contain more surface functional groups, which may play a more important role in sulfamethoxazole adsorption than surface area or hydrophobicity. Lian et al. (2014) also observed that biochar produced from Danshen residue (Chinese medicine) at low temperature (250°C) exhibited 2–7 times higher adsorption capacity to sulfamethoxazole than those prepared at higher temperatures. As can be seen, the influence of pyrolytic temperature varies among different biochar feedstocks and target pollutants. Therefore, comparative and comprehensive study of adsorption properties and the various adsorption mechanisms of various antibiotics onto biochars produced at different temperatures is of significant importance for the further application of biochar.

The objectives of this study were to further understand the adsorptive interactions of different antibiotics to biochar pyrolyzed at different temperatures. Two kinds of tetracycline and quinolone antibiotics (doxycycline and ciprofloxacin) were selected as the target contaminants. Three rice straw biochars prepared under different pyrolytic temperatures (300, 500, and 700°C) were used as the adsorbents. The adsorption isotherms and kinetics of the two antibiotics were compared, and the potential adsorption mechanisms were analyzed. The influencing factors of antibiotics adsorption including biochar dosage, pH, background electrolyte, humic acid, initial concentration, contact time, and temperature were comprehensively studied to verify the proposed adsorption mechanisms and to determine the adsorption properties under different conditions.

## Materials and methods

### Materials

Two antibiotics, doxycycline (DOX) and ciprofloxacin (CIP), were obtained from Hefei Bomei Biotechnology Co., Ltd., China. Details of the antibiotics used in this study are shown in Table S1. A stock solution was prepared in ultra-pure water (Milli-Q Millipore, 18.25 MΩ cm), and was applied to prepare the initial concentrations of the antibiotics in batch adsorption experiments. The stock and working solutions of two antibiotics were prepared daily and kept in the dark in the refrigerator. The stock solutions were diluted to get the desired concentration of working solutions in the range of 5–60 mg/L.

### Biochar preparation

The feedstock of biochar was rice straw (collected form a farm in Yiyang, Hunan province, China), which was washed and dried at 80°C for 24 h. The dried rice straw was then crushed with a comminuter to pass through a 0.145 mm sieve. The screened biomass was placed in a porcelain boat, and then heated to 300, 500 and 700°C at the rate of 5°C/min in a tube furnace (SK-1200°C, Tianjin Zhonghuan Test Electrical Furnace Co., LTD, China) and kept constant for 2 h to carbonized completely under nitrogen gas flow (60 mL/min). The biochar was then flushed with deionized water three times to remove any dirt and impurities before being oven dried for 12 h. The resulting samples were collected and stored in a plastic sealed bag in the desiccators.

### Biochar characterization

The surface properties of the biochars before and after adsorption were recorded by Fourier transforms infrared (FTIR) spectrophotometer (Nicolet 6700 spectrometer, USA) in the range of 4000–400 cm^−1^ at room temperature. The surface morphology of the biochar was observed by scanning electron microscopy (SEM, Nova Nanosem 230, USA). The Brunauer–Emmett–Teller surface area (BET) of the biochars were measured by nitrogen adsorption at 77 K using a Micromeritics TriStar II 3020. The zeta potential of biochar was measured via a zeta potential meter (Zetasizer nano-ZS90 Malvern) at 25°C. The elemental composition of the sample surface was determined using an ESCALAB 250Xi X-ray diffraction (XPS) (Thermo Fisher, USA). The X-ray diffraction (XRD) pattern was measured using a Bruker D8-Advance X-ray diffractometer (Bruker, Germany). The Raman spectrum was obtained from a Raman Microscope (Labram-010, JY, FAR).

### Adsorption experiments

All adsorption experiments were conducted by mixing the biochar with a 50 mL antibiotics solution in 100 mL plastic centrifuge tubes, and reacted in a shaker at 160 rpm. The antibiotics solution without biochar addition was set as the blank control in the following adsorption experiments. The effects of pyrolysis temperature and adsorbent dosage on the biochar adsorption ability were evaluated by adding different biochar samples of various dosages (0.2, 0.4, 0.6, 0.8, 1.2, and 1.6 g/L) into 20 mg/L of antibiotics solution. After reacting for 24 h, the suspensions were sampled and filtered through 0.45 μm Anotop syringe filters.

The effect of initial pH on the adsorption of two antibiotics by various biochars was examined at the pH range of 2–10, with initial antibiotics concentration of 40 mg/L and a biochar dosage of 0.4 g/L. The effects of competing compounds on the adsorption of these antibiotics by various biochars were investigated by adding 0.04 mol/L common coexisting ions (${\text{SO}}_{4}^{2-}$, ${\text{NO}}_{3}^{-}$, ${\text{PO}}_{4}^{3-}$, Cl^−^, Na^+^, K^+^, Ca^2+^, Mg^2+^) and 0–10 mg/L humic acid to the 40 mg/L antibiotics solutions.

Adsorption kinetics experiments were conducted by mixing 0.4 g/L of adsorbent with a 50 mL antibiotics solution (40 mg/L) in 100 mL plastic centrifuge tubes at 298 ± 1 K. The antibiotics solutions were adjusted to pH 6. The adsorbed antibiotics amount were determined after filtration of the solution at regular intervals.

Batch sorption experiments were conducted to determine the adsorption isotherm properties of antibiotics onto the biochar at 298, 308, and 318 K by mixing 0.4 g/L biochar with 50 mL antibiotics solutions (5 to 60 mg/L) in the 100 mL tubes. The plastic centrifuge tubes were shaken for 24 h. The samples were then withdrawn and filtered to measure the adsorbed antibiotics amount.

### Measurement of antibiotics

The initial and equilibrium concentrations of DOX and CIP in aqueous solution were determined by an ultraviolet spectrophotometer (UV-2550, SHIMADZU, Japan) at wavelengths of 349 and 276 nm, respectively.

### Statistical analyses

The adsorption experimental data were presented as average values of three independent replicate treatments. The adsorption data obtained in this study are presented as means ± standard deviations (SD).

## Results and discussion

### Biochar characterization

The physico-chemical characteristics of biochars prepared at different pyrolytic temperatures are shown in Table S2. The elemental compositions of biochar varied with different pyrolytic temperatures. The results demonstrated that carbon content increased at higher pyrolytic temperature, while nitrogen and oxygen content decreased with the increase of pyrolytic temperatures. This phenomenon can be clearly seen from the XPS survey spectra of biochars at 300, 500, and 700°C (Figure S1). The surface hydrophilicity of biochar may be described by the O/C ratio, since it is indicative of polar-group content (Chun et al., 2004; Chen X. et al., 2011). The lower O/C ratio of biochar produced at higher temperature indicated that the surfaces of high-temperature biochar were more aromatic and less hydrophilic due to the greater extent of carbonization and the loss of polar functional groups (Chen et al., 2008, 2012b; Ahmad et al., 2012; Kim et al., 2013). The molar (O+N)/C ratios can be used as an indicator of the polarity of carbonaceous materials. Relatively lower (O+N)/C values of BC700 (0.18) than BC500 (0.27) and BC300 (0.30) reflected a decrease of polar-group amount with increasing of pyrolytic temperature (Ahmad et al., 2012; Chen et al., 2012a,b).

The BET surface area of the biochars increased from 3.29 m^2^/g (BC300) to 9.95 m^2^/g (BC500) and 20.55 m^2^/g (BC700) with the increase of pyrolytic temperatures. The pore volume of BC700 (0.0191 cm3/g) was also much higher than that of BC300 (0.0073 cm3/g) and BC500 (0.0098 cm3/g). The average pore diameter of BC700 (6.42 nm) was smaller compared to BC300 (12.17 nm) and BC500 (7.86 nm). The volume of micropores and mesopores of BC700 accounted for a higher fraction of the total pore volume than that of BC300 and BC500 (the pore size distribution is displayed in Figure S2). The SEM results of three biochars are presented in Figure S3. The surfaces of the resulting biochars became rougher with increasing pyrolytic temperature. Moreover, more internal pores of biochar gradually emerged at higher temperature. The release of volatile components promoted the formation of vascular bundle structures in the biochar, which could improve the pore structure of biochar (Li et al., 2013). Overall, the increase in surface area and pore volume, the decrease of pore size, and the formation of internal pore structures (as a result of volatiles release during carbonization) could be observed in three biochars (Ahmad et al., 2012).

XRD patterns of different biochars are shown in Figure S4. All biochars exhibited broad peaks with 2θ at around 25°, indicating the presence of ordered graphitic structures (Tan et al., 2014; Xie et al., 2014). According to the peak intensity, the graphitization degree of the three biochars followed the order of: BC700 > BC500 > BC300. Raman spectra of different biochars show two broad bands at ~1,370 cm^−1^ (D band) and ~1,600 cm^−1^ (G band) (Figure S5). The D band originates from sp^3^ hybridization, while the G band is characteristic of crystalline graphitic/sp^2^ carbon atoms (Hussain et al., 2012; Yan et al., 2016). The ratio of G band intensity to D band intensity (*I*_G_/*I*_D_) of BC700 was higher than that of BC500 and BC300, which indicated that the ratio of disordered/ordered graphene structures was increased for BC700 (Mendonça et al., 2017).

### Effects of pyrolytic temperature and biochar dosage on antibiotics removal

**Figure 1:**
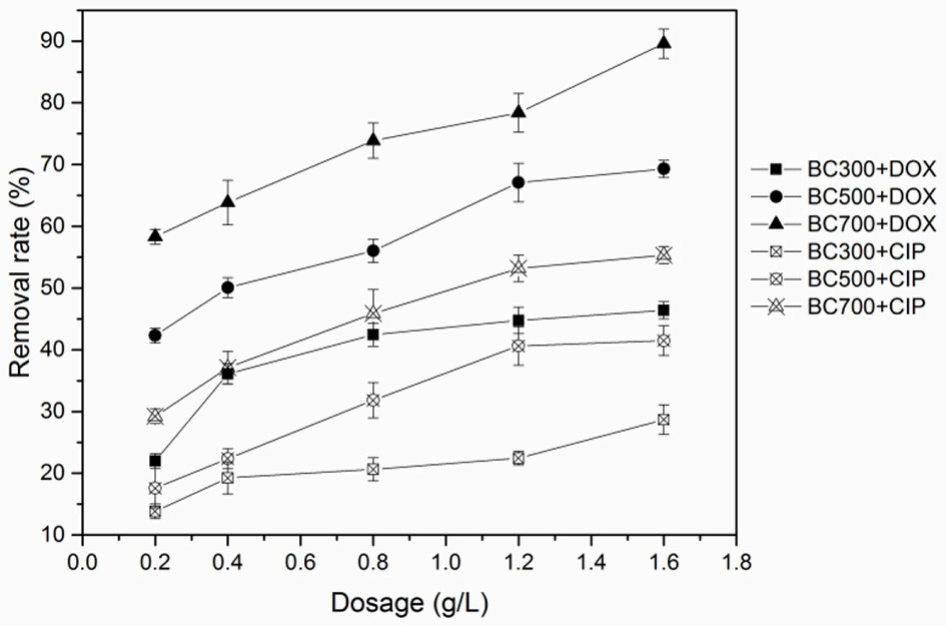
Effect of biochar dosage on the adsorption of DOX and CIP by BC300, BC500, and BC700 (initial concentration = 20 mg/L; temperature = 298 K; pH = 6; contact time = 24 h).

The effects of pyrolytic temperature and biochar dosage on antibiotics removal are shown in Figure 1. For two antibiotics, adsorption affinities among three biochars exhibited a similar trend, that is, high-temperature biochars had higher adsorption ability than the low-temperature biochars. The adsorption affinity to BC700 was significantly greater than that to BC300 and BC500. As shown in Table S2, the surface area of BC700 was markedly higher than that of BC300 and BC500. This indicated that surface area induced from the different pyrolytic temperatures played a role in adsorption. In addition, the greater volume of micropores and mesopores in BC700 might be a possible factor contributing to the greater adsorption affinity of BC700 over BC300 and BC500 (Figure S2 and Table S2). The antibiotics molecules could interact simultaneously with multiple walls of the pores in BC700 (Liu et al., 2014; Xie et al., 2014). Furthermore, the remarkably stronger adsorption to BC700 than to BC300 and BC500 seemed to be consistent with the degree of graphitization for the three biochars. The removal efficiency of two antibiotics by different biochars all trended to increase with the increasing biochar dosage (Figure 1). The more available adsorption sites on biochar promoted the removal efficiency at higher adsorbent doses.

### Effects of solution pH on antibiotics removal

**Figure 2:**
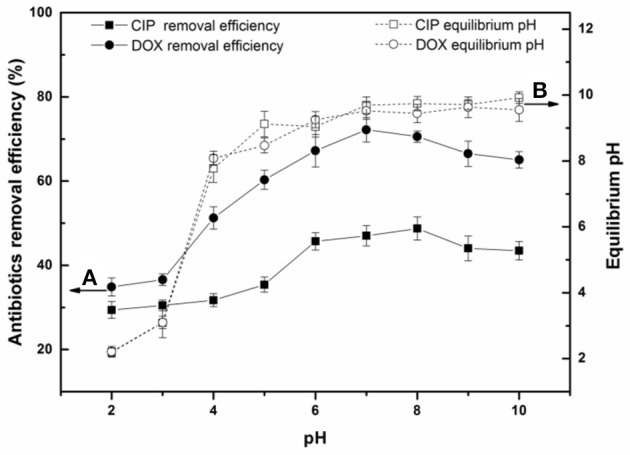
**(A)** Effect of solution pH on the adsorption of DOX and CIP by BC700; **(B)** Relationship between the initial and equilibrium pH of the sample solution (initial concentration = 40 mg/L; sorbent dose = 0.4 g/L; temperature = 298 K; contact time = 24 h).

The effects of solution pH on antibiotics removal by BC700 are shown in Figure 2. As can be seen, the removal efficiency of both antibiotics first increased and then decreased with the increase of solution pH. BC700 exhibited higher affinity for antibiotics at pH 6-8. This result suggested that the adsorption of antibiotics by biochar was highly pH-dependent, and further indicated that the graphite structures of the biochar played the predominant role in antibiotics adsorption. The variation of pH affected the surface charge of biochar and the species distribution of antibiotics in aqueous solution. The zeta-potential-pH curve of BC700 is shown in Figure S6. Biochar carried various oxygen-containing functional groups (e.g., –COOH and –OH), which may change with the different solution pH. At acidic pH, most of these functional groups are protonated and presented in positively charged form. At higher pH, the biochar surface will become negatively charged due to the deprotonation of functional groups (Tan et al., 2015). Table S1 showed the three pK_a_ values of DOX (pK_a1_ = 3.4, pK_a2_ = 7.7, pK_a3_ = 9.3; Li Z. et al., 2015) and two pK_a_ values of CIP (pK_a1_ = 6.2, pK_a2_ = 8.8; Zhong et al., 2015). The speciation of DOX and CIP varied with different pH conditions and were shown in Figure S7. At alkaline pH, the deprotonated form of antibiotics were anionic species. The dominant form of antibiotics would be zwitterionic and cationic species at neutral and lower pH, which were both available and effective π-electron-acceptors, which could induce strong π-π EDA interaction with the graphite structures of the biochar. With increasing pH, deprotonation of the antibiotics group can significantly decrease the π-withdrawing ability of the group and can suppress the interaction force (Ji et al., 2009; Huang et al., 2017). In addition, the electrostatic repulsion between the anionic antibiotics and the deprotonated functional groups of biochar increased with increasing pH, which might induce the decrease of adsorption ability. The pH value of the reactive solution increased sharply from initial solution pH 2 to 4, and remained near 10 after the equilibrium was reached due to the buffering capacity of the biochar, which might be attributed to the release of alkaline substances in the biochar (Zhou et al., 2016).

### Effect of background electrolyte and HAs

**Figure 3:**
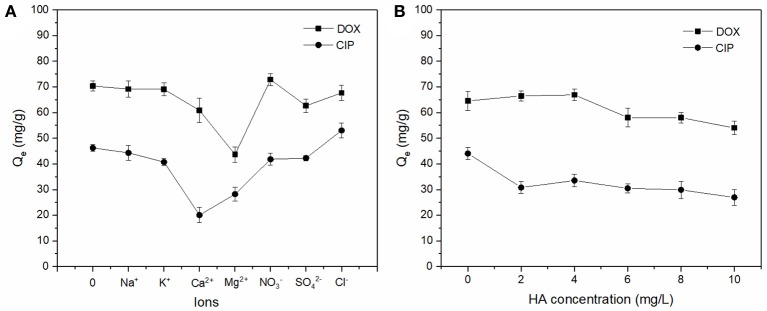
Effect of background electrolyte **(A)** and HA **(B)** on the adsorption of DOX and CIP by BC700 (initial concentration = 40 mg/L; ions concentration = 0.04 mol/L; sorbent dose = 0.4 g/L; temperature = 298 K; contact time = 24 h; pH = 6).

The effect of background electrolyte and HA on DOX and CIP adsorption by BC700 are shown in Figure 3A. It was found that the monovalent cations (Na^+^ and K^+^) exhibited lower influence on the adsorption capacity of antibiotics than that of divalent ions (Mg^2+^ and Ca^2+^). This could be attributed to the fact that the divalent ions had stronger squeezing-out effect due to their high polarizing power (Jiang et al., 2016). The adsorbed Mg^2+^ and Ca^2+^ might have hydration shells of dense water, which could hinder the available adsorption sites by blocking the hydrophobic adsorption region. In addition, Mg^2+^ and Ca^2+^ might directly compete for adsorption sites due to inner-sphere complexation, and therefore hinders the formation of charge-assisted H-bonds with antibiotics (Xie et al., 2014; Peiris et al., 2017). The effect of background electrolyte anions (Cl^−^, NO^3−^, and ${\text{SO}}_{4}^{2-}$) on antibiotics adsorption by BC700 was not significant, which could be explained in that the interaction between anions and the negatively charged biochar surface possessed strong repellency and therefore low interference on antibiotics adsorption.

The effect of Humic acids (HAs) on adsorption process is shown in Figure 3B. The inhibition effects were observed in the adsorption of both antibiotics. HAs contain numerous polar functional groups, including carboxyl, phenolic, carbonyl, and amino (Lian et al., 2015; Peiris et al., 2017), which made them strongly interact with the polar fraction of the biochar via H bonding and with the hydrophobic fraction by π-π electron donor acceptor (EDA) interactions (Peiris et al., 2017). In addition, HAs might combine with antibiotics to form soluble complexes to reduce the adsorption capacity (Hu et al., 2016; Jiang et al., 2016). Moreover, it was reported that HAs mitigated adsorption due to the blocking of pores, and therefore reducing access to adsorption sites of biochar at pH of ~6 (Xie et al., 2014).

### Adsorption kinetics

Figure S8 presents the effect of contact time on the CIP and DOX adsorption. It can be seen that the adsorption of CIP and DOX both rapidly increased in the first 2 h, which was due to the presence of abundant and available active sites on the biochar surface. Then the uptake of two antibiotics slowly increased until the adsorption equilibrium was reached. To further investigate the adsorption mechanism, pseudo-first-order, and pseudo-second-order models were applied to analyze the experimental result. The equations of two models are expressed as follows (Jiang et al., 2016):

(1) $$\begin{array}{r} \ln\;\left(q_{\text{e}}-q_{\text{t}}\right)=\ln\;q_{\text{e}}-k_{1}t \end{array}$$

(2) $$\begin{array}{r} \frac{t}{q_{\text{t}}}=\frac{1}{k_{2}q_{\text{e}}^{2}}+\frac{t}{q_{\text{e}}} \end{array}$$

where *q*_e_ and *q*_t_ (mg g^−1^) represent the adsorption amount of antibiotics at equilibrium and at time *t, k*_1_ (min^−1^) and *k*_2_ (g mg^−1^ min^−1^) are the reacted rate constant of two models, respectively.

**Figure 4:**
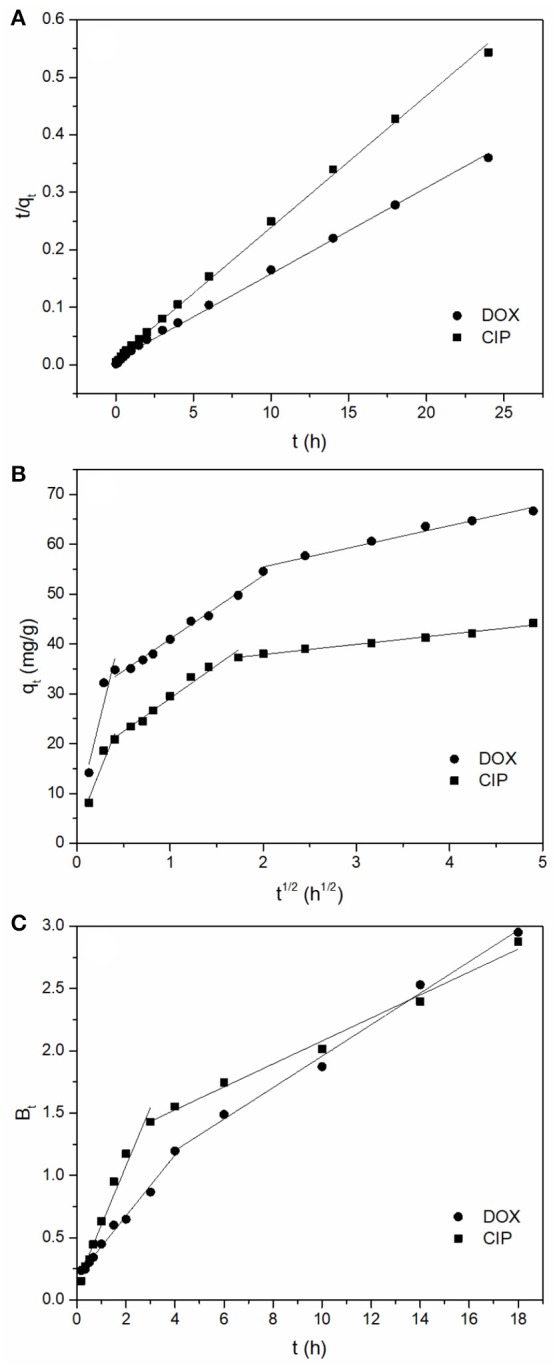
**(A)** Pseudo-second-order plots for antibiotics adsorption, **(B)** Intra-particle diffusion plots for antibiotics adsorption, **(C)** Boyd plots for antibiotics adsorption (initial concentration = 40 mg/L; sorbent dose = 0.4 g/L; temperature = 298 K; pH = 6).

The results are presented in Figure 4 and the calculated parameters are listed in Table 1. The data of adsorption of DOX and CIP both fitted better to pseudo-second-order model (*R*^2^ = 0.998 and 0.997) than to the pseudo-first-order model. This can be further confirmed by the small discrepancy between the calculated *q*_e_ value and the experimental results. The better fit of adsorption process by the pseudo-second-order model suggested that the chemisorption may be the rate-limiting mechanism of DOX and CIP adsorption. Thus, it was inferred that DOX and CIP were mainly adsorbed onto the surface of BC700 by chemical interactions, such as the hydrogen bonding and π-π EDA interaction.

**Table 1:** The model parameters and the corresponding correlation coefficient of kinetics models.

**Kinetics**	**Parameters**	**DOX**	**CIP**
Pseudo-first-order	*q*_e_ (mg/g)	53.35	38.17
	*K*_1_ (1/min)	4.35	2.75
	*R*^2^	0.795	0.840
Pseudo-second-order	*q*_e_ (mg/g)	67.11	43.67
	*K*_2_ (g/mg min)	0.026	0.054
	*R*^2^	0.997	0.998

In order to further determine the diffusion mechanisms and to identify the possible rate controlling procedures, an intra-particle diffusion model was adopted. The parameters of intra-particle diffusion are usually analyzed by the following equation (Wu et al., 2014):

(3) $$\begin{array}{r} q_{\text{t}}=k_{\text{id}}{\text{t}}^{1/2}+c_{\text{i}} \end{array}$$

where *q*_t_ (mg g^−1^) represents the adsorption amount of antibiotics at time *t, k*_id_ is the intra-particle diffusion rate constant (mg/g·min^1/2^), and *c*_i_ is the intercept related to the thickness of the boundary layer.

As shown in Figure 4B, there were three linear portions in the plots of *q*_t_ against *t*^1/2^, which suggested that the adsorption process included multiple steps. The calculated values of *c*_i_ were not zero (Table S3), indicating that intra-particle diffusion was involved in the diffusion process and it was one of the rate limiting steps in the adsorption process (Hu et al., 2011). During the initial reaction process, antibiotics molecules were captured by the exterior surface of the BC700, therefore, the adsorption process was firstly controlled by film diffusion (Abel et al., 2017). Then, the antibiotics molecules further passed into the pores of BC700 and were subsequently captured by the interior surfaces, such that the adsorption of antibiotics onto the BC700 was controlled by intra-particle diffusion (Ai et al., 2011; Wu et al., 2014; Abel et al., 2017).

To determine the actual rate-controlling step of the overall antibiotics adsorption process, the Boyd kinetic model were further applied to analyze the adsorption kinetic result (Boyd et al., 1947; Abel et al., 2017), which is expressed as follows:

(4) $$\begin{array}{r} F=1-\frac{6}{\pi^{2}}\exp\left(-B_{t}\right) \end{array}$$

where *F* is the fraction of antibiotics adsorbed at different time *t*. *B*_t_ is a mathematical function of *F*, which is given by:

(5) $$\begin{array}{r} F=\frac{q_{t}}{q_{e}} \end{array}$$

where *q*_t_ and *q*_e_ are the adsorption quantities at time *t* and equilibrium, respectively. The kinetic expression Equation (4) can be represented as:

(6) $$\begin{array}{r} B_{t}=-0.4977-\ln\;\left(1-F\right) \end{array}$$

As shown in Figure 4C, there were two linear portions in the plots of *B*_t_ vs. *t* for the adsorption of antibiotics onto BC700 and all lines did not pass through the origin (Table S4). Based on the analysis of the plot of this model, it was suggested that the film diffusion initially controlled the adsorption process, and intra-particle diffusion mechanisms took over subsequently (Wang et al., 2016).

### Adsorption isotherms

**Figure 5:**
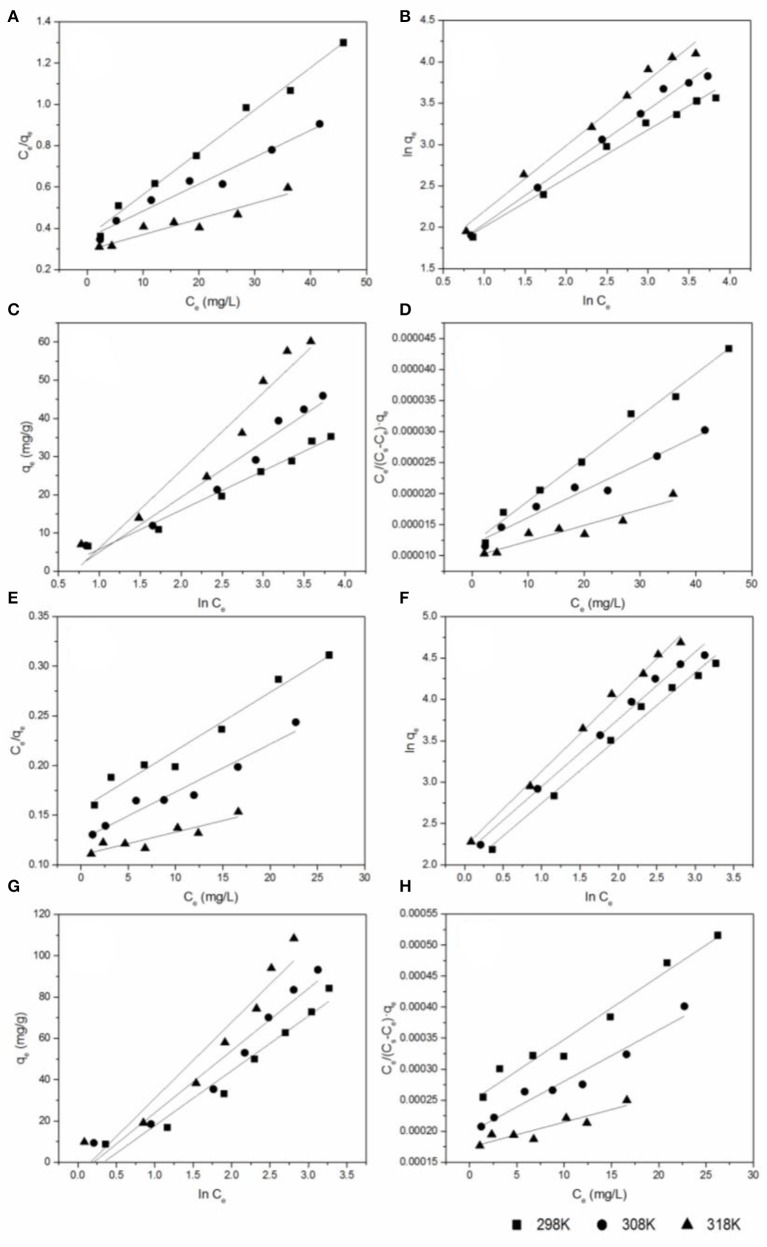
The equilibrium isotherms for antibiotics adsorbed by BC700: **(A)** the Langmuir model of CIP; **(B)** the Freundlich model of CIP; **(C)** the Temkin model of CIP; **(D)** the BET model of CIP; **(E)** the Langmuir model of DOX; **(F)** the Freundlich model of DOX; **(G)** the Temkin model of DOX; **(H)** the BET model of DOX (sorbent dose = 0.4 g/L; pH = 6; contact time = 24 h).

Figure S9 shows the effect of initial antibiotic concentration on the adsorption capacity at different temperatures (298, 308, and 318 K). It can be observed that the adsorption capacity of CIP and DOX increased from 6.57 to 60.18 mg/g and 8.93 to 108.42 mg/g, respectively, with the initial antibiotic concentration increased in the range of 5–60 mg/L. The higher initial antibiotic concentration will increase the driving force and provide more chance for antibiotic molecule to be captured by biochar (Jiang et al., 2016). Four adsorption isothermal models including Langmuir, Freundlich, Tempkin, and BET models were applied to fit the experimental data (Figure 5). These adsorption models are expressed by the following equations (Hu et al., 2011; Wang et al., 2016):

(7) $$\begin{array}{r} \frac{C_{\text{e}}}{q_{\text{e}}}=\frac{C_{\text{e}}}{q_{\max}}+\frac{1}{q_{\max}K_{\text{L}}} \end{array}$$

(8) $$\begin{array}{r} \ln\;q_{\text{e}}=\ln\;K_{\text{F}}+\frac{1}{n}\ln\;C_{\text{e}} \end{array}$$

(9) $$\begin{array}{r} q_{\text{e}}=B_{\text{T}}\ln\;K_{\text{T}}+B_{\text{T}}\ln\;C_{\text{e}} \end{array}$$

(10) $$\begin{array}{r} \frac{C_{\text{e}}}{\left(C_{s}-C_{\text{e}}\right)q_{\text{e}}}=\frac{1}{K_{\text{b}}q_{m}}+\frac{C_{\text{e}}\left(K_{\text{b}}-1\right)}{K_{\text{b}}q_{m}C_{\text{s}}} \end{array}$$

where *q*_e_ is the amount of the antibiotics adsorbed (mg/g), *q*_max_ is the maximum adsorption capacity (mg/g), *C*_e_ is the equilibrium concentration of solution (mg/L), and *C*_s_ is saturation concentration of solute (mg/L). *K*_L_ (L/mg) is the Langmuir constant related to the affinity, which is used to indicate whether the adsorption equilibrium is favorable (0 < *K*_L_ < 1) or unfavorable (*K*_L_ >1). *K*_F_ (L/mg) and *n* are the Freundlich constants, which indicate the adsorption capacity and intensity, respectively. *B*_T_ = *RT*/*b*_T_ and *b*_T_ (J/mol) is the Temkin constant related to the heat of adsorption, *R* is the universal gas constant (8.314 J/mol.K), *T* is the temperature (K), and *K*_T_ (L/mg) is the maximum binding energy constant. *K*_b_ is the BET constant.

The Langmuir model assumes monolayer coverage of adsorbed molecules on a homogeneous surface of adsorbent (Sun et al., 2014). The Freundlich isotherm is an empirical equation assuming that the adsorption process takes place on heterogeneous surfaces, which is not restricted to the formation of a monolayer (Sun et al., 2014). The Temkin isotherm was based on an assumption that there are indirect interactions between adsorbate molecules, and the heat of adsorption decreases linearly with surface coverage (Njoku et al., 2014). The BET adsorption model assumes that a multilayer on the adsorbent surface in a random distribution of adsorbate is formed (Hussain et al., 2013).

The equilibrium isotherms for two antibiotics adsorbed by BC700 are shown in Figure 5. The values of parameters and the corresponding correlation coefficients of isotherm models are shown in Table 2. The maximum adsorption capacity (*q*_max_) was found to be in the following order: DOX > CIP. The *q*_max_ of DOX and CIP from Langmuir model was 170.36 to 432.90 mg/g and 48.80 to 131.58 mg/g, respectively, as the solution temperature increased from 298 K to 318 K. The maximum adsorption capacities of various adsorbents for CIP and DOX are listed in Table S5. As can be seen, the DOX and CIP adsorption capacity of BC700 is higher or comparable than some adsorbents reported previously, suggesting that BC700 may be an effective adsorbent for DOX and CIP removal from contaminated water. The experimental data of DOX and CIP exhibited higher correlation with the Freundlich model, with the correlation coefficients *R*^2^ higher than 0.98 regarding the three studied temperatures, thus indicating that the adsorption of the two antibiotics probably took place onto the heterogeneous surfaces of BC700. This result is consistent with the kinetics results, i.e., that the adsorption of DOX and CIP on the BC700 could be governed by multiple mechanisms. The 1/*n* < 1 suggested that the adsorption of DOX and CIP onto BC700 was favorable. The increase of *K*_F_ with increasing temperature implied that high temperatures favored adsorption, and the adsorption was endothermic in nature. The *K*_F_ of the adsorption of DOX (7.07–9.22) onto BC700 were much higher than that of CIP (3.85–4.14), suggesting that BC700 showed higher affinity for DOX.

**Table 2:** The model parameters and the corresponding correlation coefficient of isotherm models.

**Isotherms**	**Parameters**	**DOX**			**CIP**		
		**298 K**	**308 K**	**318 K**	**298 K**	**308 K**	**318 K**
Langmuir	*q*_max_ (mg/g)	170.36	207.90	432.90	48.80	76.69	131.58
	*K*_l_ (L/mg)	0.038	0.038	0.021	0.057	0.037	0.026
	*R*^2^	0.96	0.94	0.89	0.988	0.96	0.88
Freundlich	1/*n*	0.79	0.81	0.91	0.69	0.79	0.59
	*K*_F_ (L/mg)	7.07	8.44	9.22	3.85	4.05	4.14
	*R*^2^	0.987	0.990	0.994	0.990	0.985	0.984
Tempkin	*K*_T_ (L/mg)	0.72	0.82	0.85	0.65	0.52	0.49
	*B*_T_	26.54	26.54	26.54	10.24	14.37	20.33
	*R*^2^	0.948	0.936	0.915	0.945	0.974	0.928
	*b*_T_ (J/mol)	93.34	85.11	71.74	241.83	178.17	130.07
BET	*K*_b_	26.97	27.31	15.70	1720.40	1,110.89	779.64
	*q*_m_ (mg/g)	150.53	185.09	365.83	48.66	76.43	131.03
	*R*^2^	0.965	0.946	0.919	0.988	0.961	0.884

### Possible mechanisms for DOX and CIP adsorption

**Figure 6:**
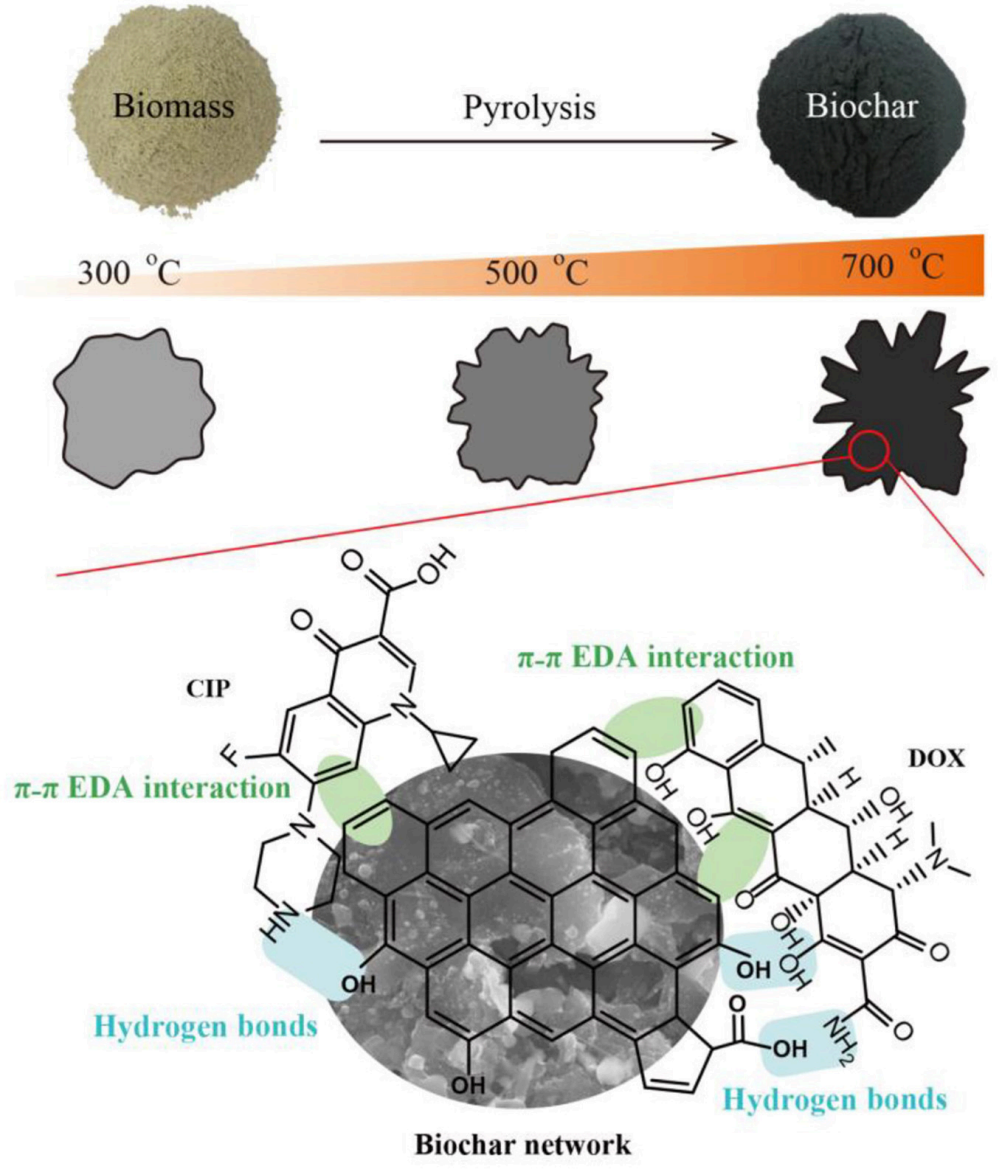
The proposed mechanisms for DOX and CIP adsorption.

The FTIR spectra of BC700 before and after adsorption of DOX and CIP are displayed in Figure S10. Typical absorption peaks of biochar existed in the spectra of BC700, including 3430 cm^−1^ (phenolic O–H stretching) (Xu et al., 2011), 1620 cm^−1^ (the stretching vibration of aromatic C = C bonds) (Chen B. et al., 2011; Li M. et al., 2015), 1390 cm^−1^ (–COO– symmetric stretching) (Tang et al., 2011), and 1090 cm^−1^ (alkoxy C–O–C bonds) (Zhang et al., 2012). A series of peaks at 400–800 cm^−1^ were formed after the adsorption of DOX and CIP, which were attributed to aromatic –CH stretching vibration, C–C stretch or C–O–H bending of antibiotics (Ahmed et al., 2016), suggesting that plenty of antibiotics molecules were adsorbed onto the surface of the biochar. The peak of the O–H stretching shifted from 3,430 cm^−1^ to 3,440 cm^−1^, the peaks of –COO– symmetric stretching shifted from 1,390 to 1,430 cm^−1^, and the alkoxy C–O–C bonds shifted from 1,090 cm^−1^ to 1,100 cm^−1^ after adsorption of two antibiotics. These results suggested that the oxygen-containing functional groups of BC700 (including hydroxyl, carboxyl, and alkoxy groups) might participate in the adsorption of antibiotics by forming hydrogen bonding between the phenolic hydroxyl, carboxyl, and amino groups of DOX and CIP. The peaks of aromatic C = C bonds also shifted from 1620 to 1630 cm^−1^ after adsorption of the antibiotics, indicating that the π-π EDA interaction between antibiotics and BC700 was proposed as the significant force for DOX and CIP adsorption (Jiang et al., 2016). The proposed mechanisms for DOX and CIP adsorption are illustrated in Figure 6.

The surface area, pore volume, and surface functional groups of the biochar changed with the increase of pyrolytic temperature, thereby enhancing the adsorption efficiency of DOX and CIP. The higher adsorption ability is consistent with the higher surface area and the greater volume of micropores and mesopores of BC700. This indicated that surface area and pore volume contributed to the greater adsorption affinity of BC700 as more favorable adsorption sites were available due to the pore-filling effect. Furthermore, the remarkably stronger adsorption to BC700 than to BC300 and BC500 seemed to be consistent with the change of surface properties of the three biochars. The hydrogen bonding of high-temperature biochar might be strengthened by the condensed aromatic surfaces, as the hydrogen bonding to functional groups of BC700 was facilitated by the large π subunit of its aromatic substrate (Fang et al., 2014; Yan et al., 2016). The π-π EDA interaction was also the primary mechanism governing DOX and CIP adsorption by biochar. The two antibiotics could interact more strongly with higher graphitic surfaces of BC700 via strong π-π EDA interaction. It was observed that BC700 exhibited higher affinity for DOX than for CIP. DOX contained more aromatic rings and phenolic hydroxyl than CIP, which might provide more opportunities for DOX to be captured by BC700 through hydrogen bonding or π-π EDA interaction. In addition, the water solubility of DOX (630 mg/L) was much less than that of CIP (30,000 mg/L); therefore, BC700 provided a more hydrophobic microenvironment to accommodate weakly hydrophilic DOX, which resulted in the higher adsorption efficiency for DOX.

## Conclusions

Three rice straw biochars prepared under different pyrolytic temperatures exhibited strong adsorption to two kinds of antibiotics (doxycycline and ciprofloxacin). The removal efficiency was greatly affected by the solution pH, background electrolyte, and humic acid. The high-temperature biochar (BC700) had higher adsorption capacity for the two antibiotics, which was consistent with its higher surface area, greater volume of micropores and mesopores, and higher graphitic surfaces. In addition, BC700 showed higher adsorption affinity for DOX than for CIP. The π-π EDA interaction and hydrogen bonding might be the predominant adsorption mechanisms leading to the high affinity of BC700 for antibiotics. Overall, BC700 would be a cost-effective and promising adsorbent for doxycycline and ciprofloxacin removal.

## Author contributions

ZZ, XT, YL, ST, GZ, and LJ: Contributed to the experiment operation, data analysis, and draft manuscript writing; ZY, NL, SL, and JL: Contributed to the planning and design of the project paper.

### Conflict of interest statement

The authors declare that the research was conducted in the absence of any commercial or financial relationships that could be construed as a potential conflict of interest.
