# Supplementary material for: Comprehensive Adsorption Studies of Doxycycline and Ciprofloxacin Antibiotics by Biochars Prepared at Different Temperatures
Source: Front Chem. 2018 Mar 27;6:80. doi: 10.3389/fchem.2018.00080 (PMC5880934; doi:10.3389/fchem.2018.00080)
Supplement: Supplementary file 1 [file Presentation1.pdf]

Supplementary Information for:

Comprehensive adsorption studies of doxycycline and ciprofloxacin antibiotics by biochars prepared at different temperatures

Zhiwei Zeng[a, b, c], Xiaofei Tan[a, b, *], Yunguo Liu[a, b, *], Sirong Tian[a, b], Guangming Zeng[a, b], Luhua Jiang[a, b], Shaobo Liu[d, e], Jiang Li[a, b], Ni Liu[a, b], Zhihong Yin[a, b]

[a] College of Environmental Science and Engineering, Hunan University, Changsha 410082, P.R. China

[b] Key Laboratory of Environmental Biology and Pollution Control (Hunan University), Ministry of Education, Changsha 410082, P.R. China

[c] College of Architecture and Urban Planning, Hunan City University, Yiyang 413000, P.R. China

[d] School of Metallurgy and Environment, Central South University, Changsha 410083, PR China

[e] College of Environmental Science and Engineering Research, Central South University of Forestry and Technology, Changsha 410004, PR China

* Correspondence: Xiaofei Tan, tanxf@hnu.edu.cn; Yunguo Liu, hnuliuyunguo@gmail.com

16 pages, 5 tables, and 10 figures.

Table S1. Details of the tetracycline hydrochloride used in this study

	Doxycycline	Ciprofloxacin
IUPAC name	(4S,4aR,5S,5aR,6R,12aR)-4-(dimethylamino)-1,5,10,11,12a-pentahydroxy-6-methyl-3,12-dioxo-4a,5,5a,6-tetrahydro-4H-tetracene-2-carboxamide	1-cyclopropyl-6-fluoro-4-oxo-7-piperazin-1-ylquinoline-3-carboxylic acid
Commercial name	Doxycycline	Ciprofloxacin
CAS number	564-25-0	85721-33-1
Appearance	Yellow crystalline powder	Faint to light yellow crystalline powder
Molecular Formula	$C_{22}H_{24}N_2O_8$	$C_{17}H_{18}FN_3O_3$
Molecular weight	444.4 g/mol	331.3 g/mol
Melting point	201 ℃	255-257 ℃
Water Solubility	630 mg/L	30000 mg/L
pK_a	pK_{a1} = 3.4, pK_{a2} = 7.7, pK_{a3} = 9.3	pK_{a1} = 6.2, pK_{a2} = 8.8
Molecular structure	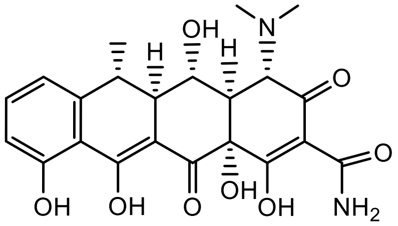	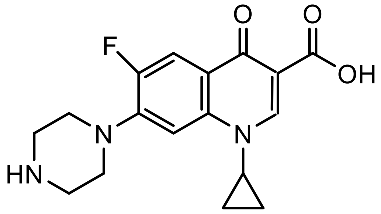

Table S2. Physico-chemical characteristics of biochars produced at different pyrolysis temperatures

Biochar	Elemental composition (%, mass based) [a]					Atomic ratios		BET surface area (m^2/g)	Average pore diameter (nm)	Pore Volume (cm^3/g)	Micropore and mesopore volume (cm^3/g)
	C	N	O	P	S	O/C	(N+O)/C				
BC300	70.75	2.5	25.18	0.93	0.65	0.27	0.30	3.29	12.17	0.0073	0.0044
BC500	72.57	2.52	23.69	0.72	0.5	0.24	0.27	9.95	7.86	0.0098	0.0069
BC700	80.48	2.32	16.38	0.47	0.36	0.15	0.18	20.55	6.42	0.0191	0.0146

[a] Determined by X-ray photoelectron spectroscopy (XPS).

Table S3. Intra-particle diffusion parameters for the adsorption of antibiotics by BC700.

	Sections	k_{id} (mg/g·$h^{0.5}$)	c_i	R_i^2
CIP	Section1	46.64	3.02	0.857
	Section2	13.25	15.83	0.974
	Section3	2.04	33.80	0.987
DOX	Section1	76.24	6.06	0.797
	Section2	12.74	28.24	0.986
	Section3	4.13	47.19	0.970

Table S4. Boyd plot parameters for the adsorption of antibiotics by BC700.

	Sections	k_{id}	c_i	R_i^2
CIP	Section1	0.47	0.13	0.967
	Section2	0.092	1.16	0.990
DOX	Section1	0.24	0.19	0.989
	Section2	0.13	0.69	0.992

Table S5. Maximum adsorption capacities of various adsorbents for CIP and DOX.

	Adsorbent	Temperature (K)	q_{max} (mg/g)	References
CIP	rice straw biochar (700 ℃)	298	48.80	In this study
	magnetic herbal biochar		47.62	(Kong et al., 2017)
	biocomposite fibers of graphene oxide/calcium alginate		66.25	(Wu et al., 2013)
	magnetic carbon composite	303	90.1	(Mao et al., 2016)
DOX	rice straw biochar (700 ℃)	298	170.36	In this study
	Cu(II) impregnated biochar	298	52.374	(Liu et al., 2017)
	graphene nanosheet	297.15	110	(Rostamian & Behnejad, 2018)
	NaY zeolite	303	252.12	(Ali & Ahmed, 2017)

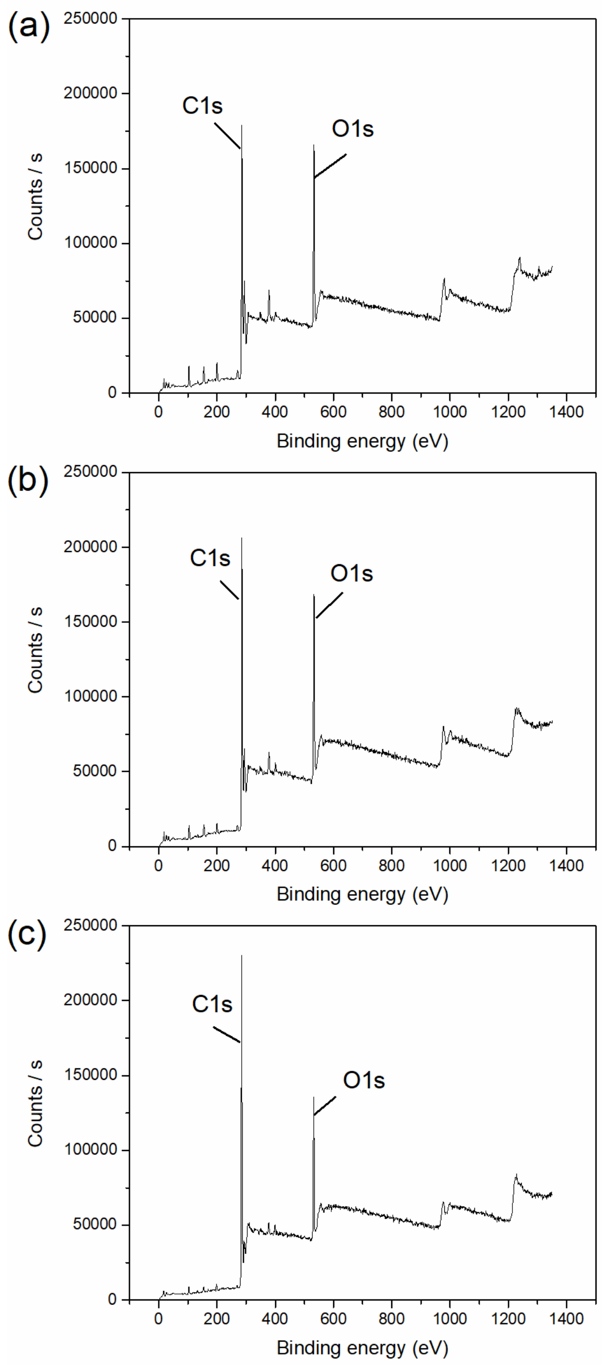

Fig. S1. XPS survey spectra of biochar produced at (a) 300 ℃, (b) 500 ℃, and (c) 700 ℃.

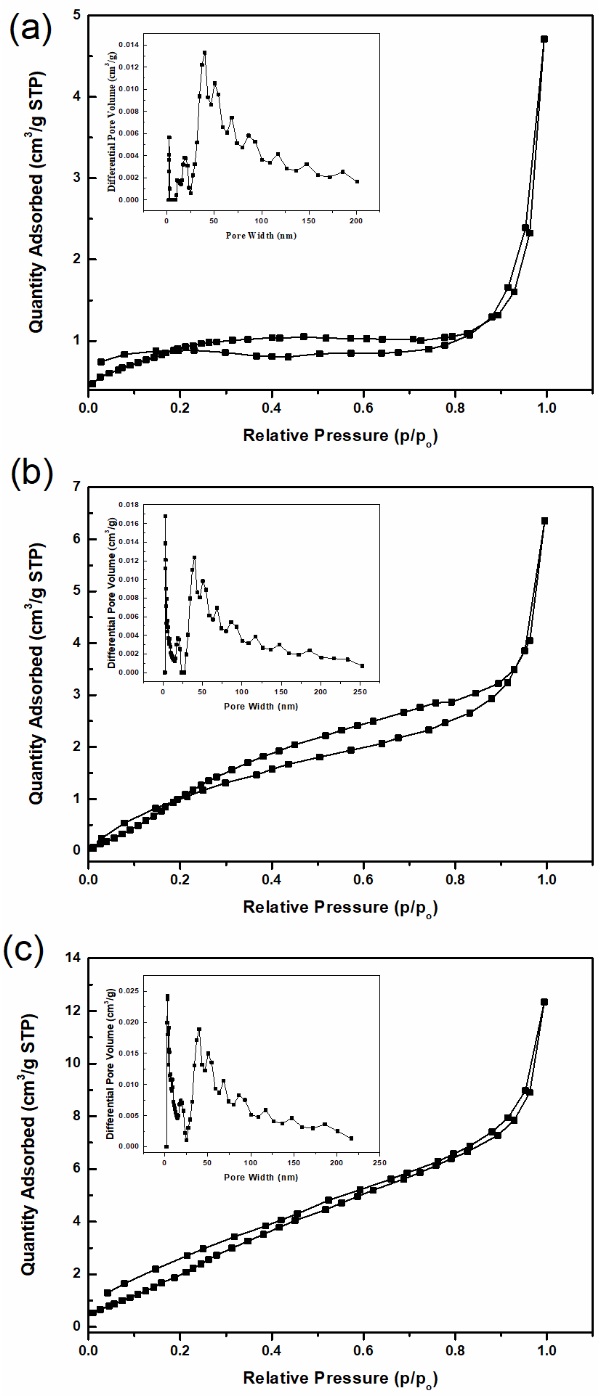

Fig. S2. The nitrogen adsorption-desorption isotherms and pore size distribution of (a) BC300, (b) BC500, and (c) BC700.

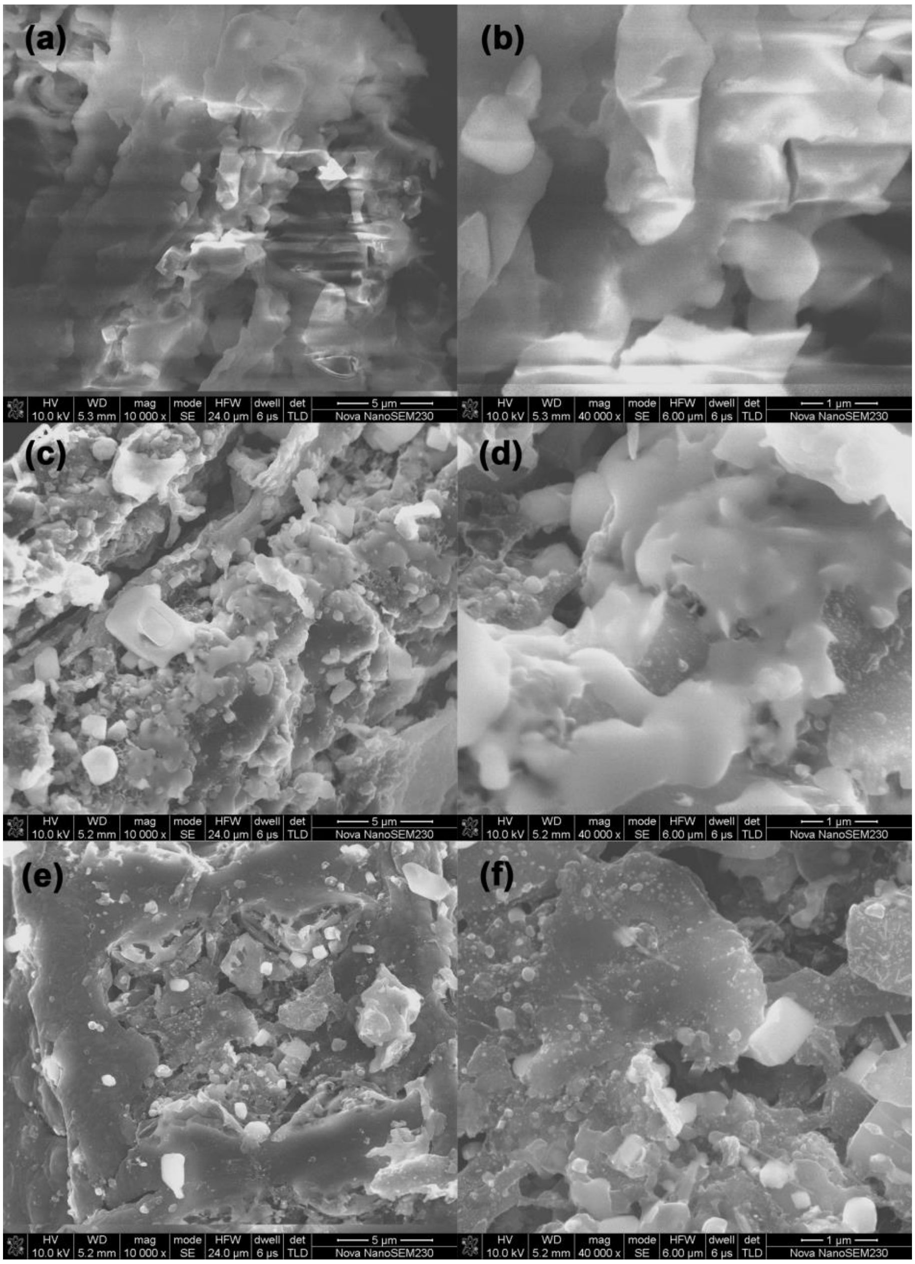

Fig. S3. SEM micrograph results of (a) BC300, (b) BC500, and (c) BC700.

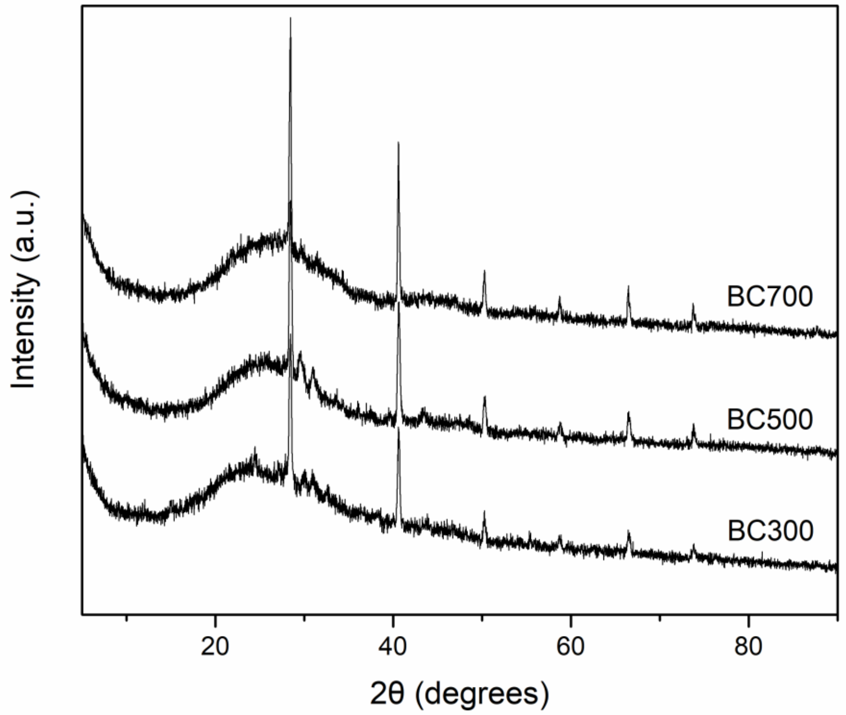

Fig. S4. X-ray diffraction (XRD) patterns of different biochars.

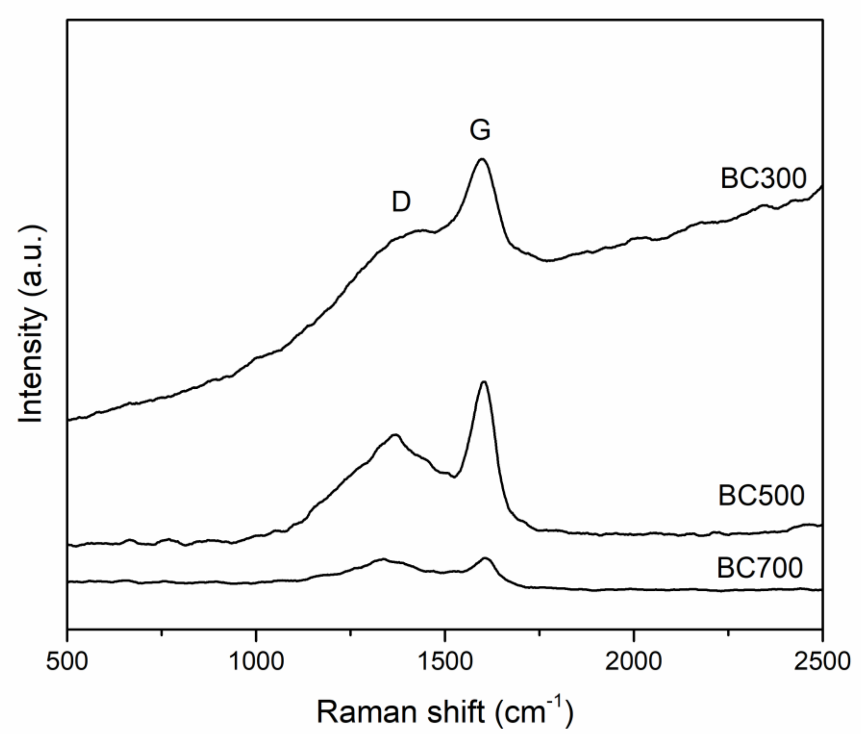

Fig. S5. Raman spectra of different biochars.

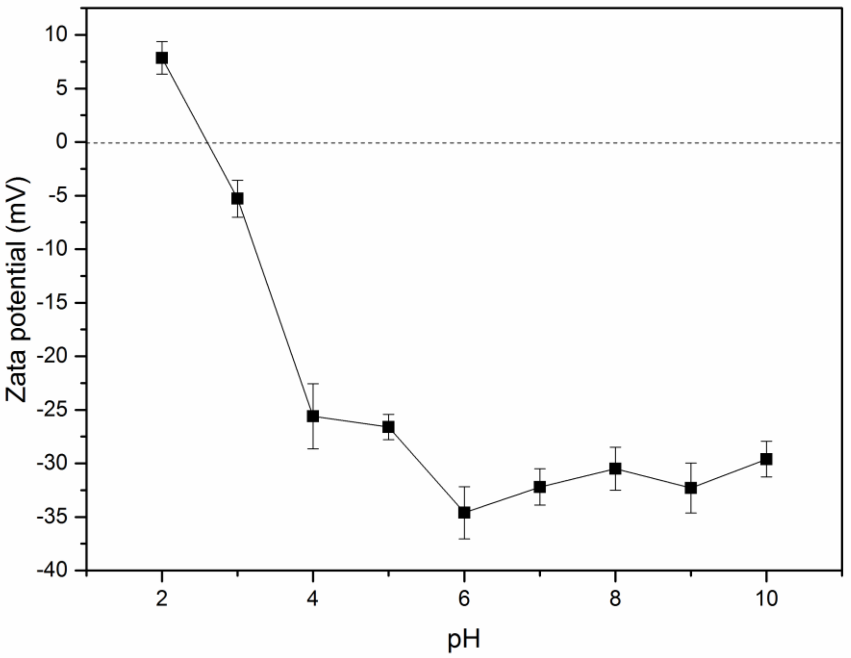

Fig. S6. The zeta-potential-pH curves of BC700**.**

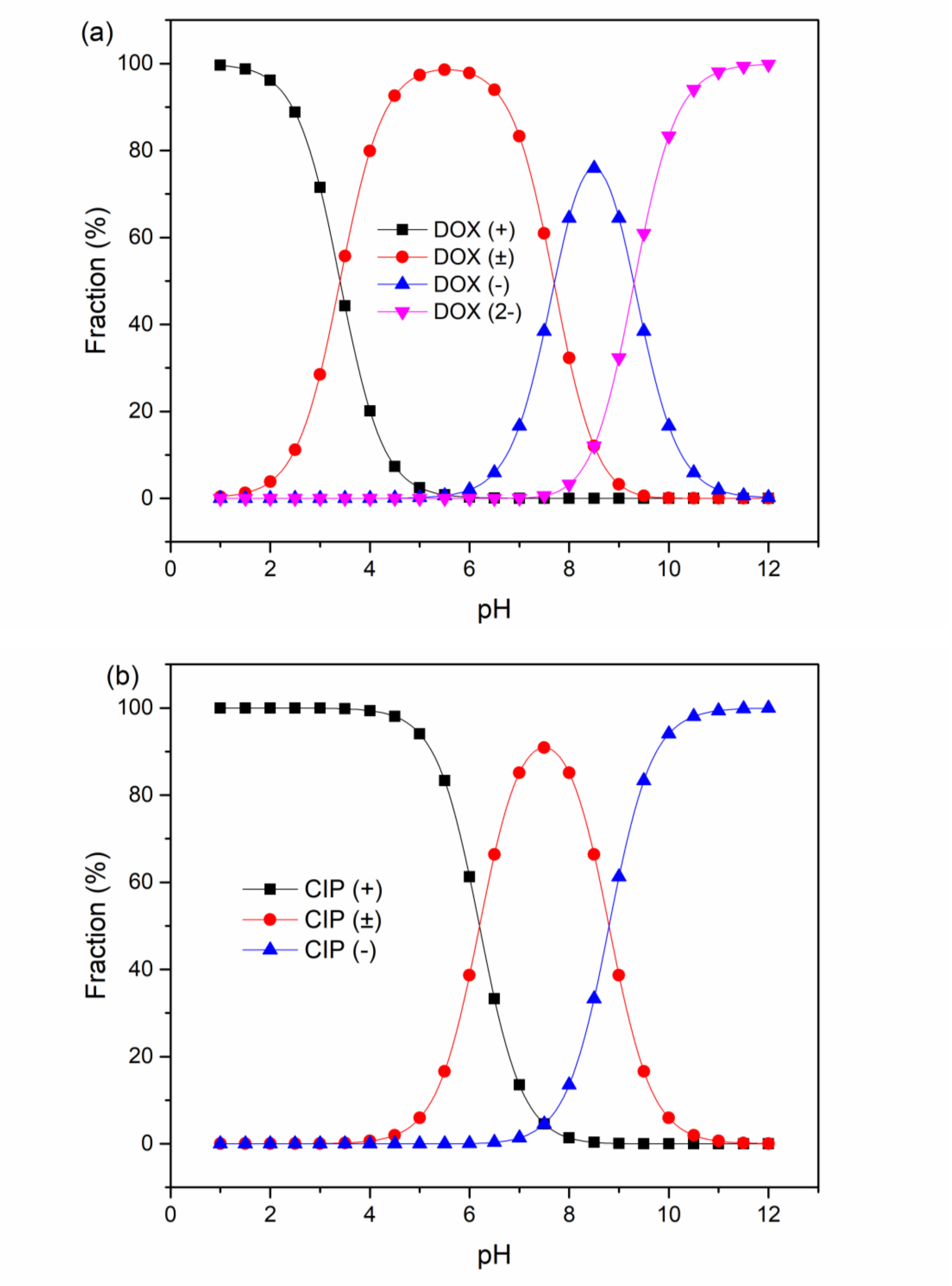

Fig. S7. The speciation of DOX (a) and CIP (b) under different pH conditions.

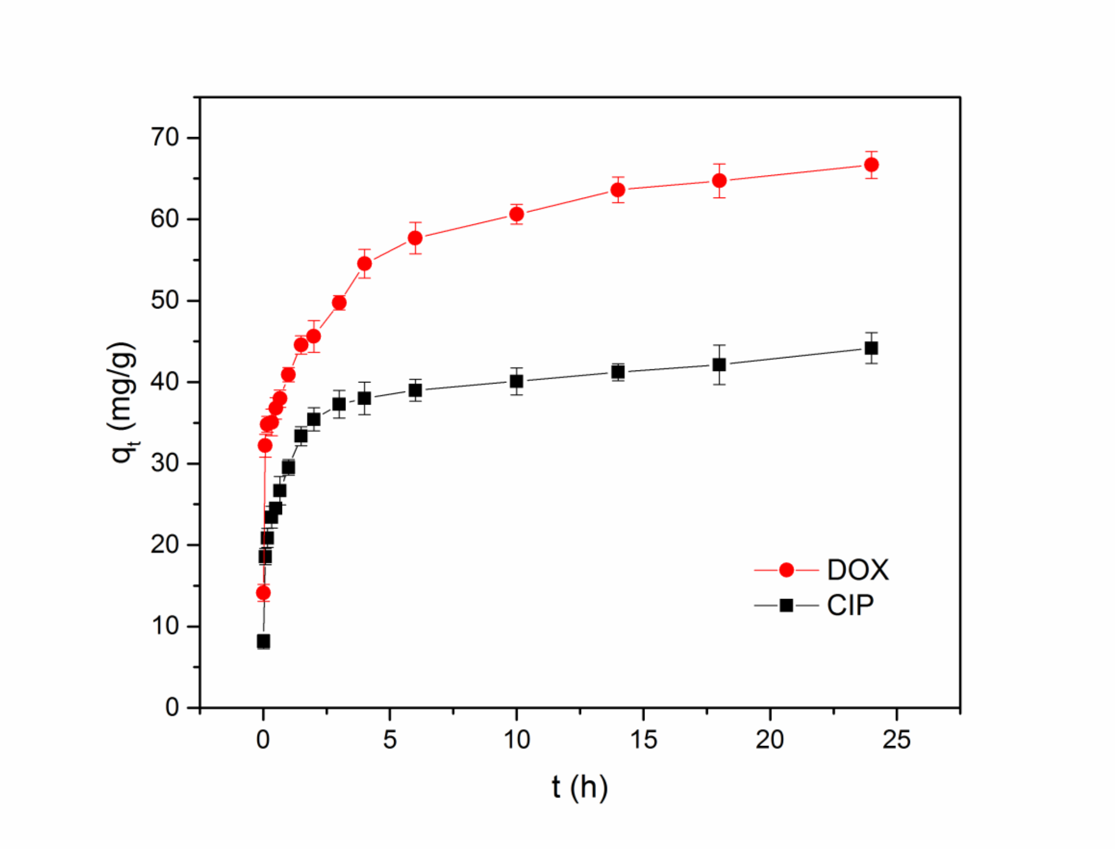

Fig. S8. The effect of contact time on CIP and DOX adsorption.

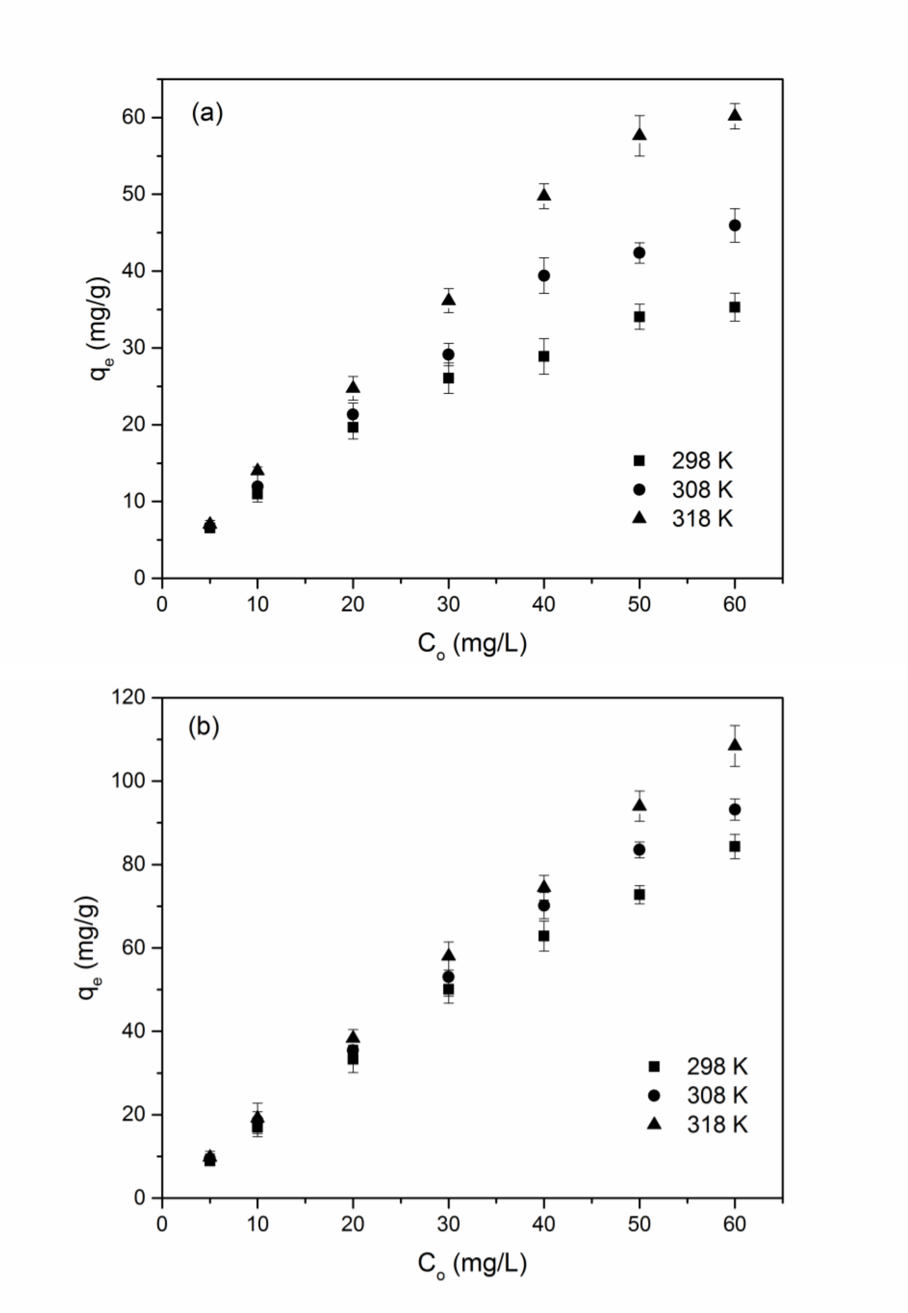

Fig. S9. The effect of antibiotic concentration on CIP (a) and DOX (b) adsorption.

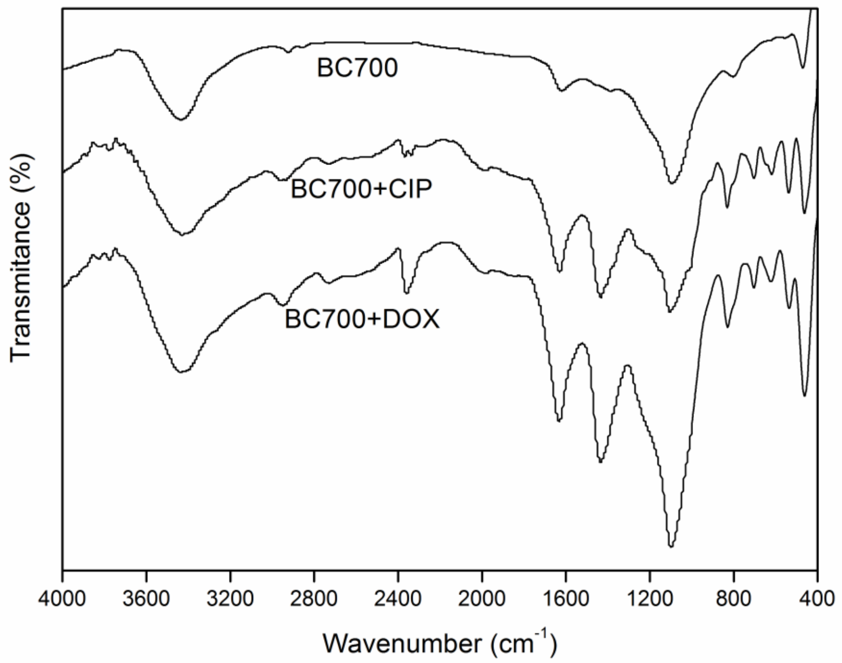

Fig. S10. The FTIR spectra of BC700 before and after adsorption of DOX and CIP.

Reference

Ali, M.M.M., Ahmed, M.J. 2017. Adsorption behavior of doxycycline antibiotic on NaY zeolite from wheat (Triticum aestivum) straws ash. *Journal of the Taiwan Institute of Chemical Engineers*, **81**, 218-224.

Kong, X., Liu, Y., Pi, J., Li, W., Liao, Q., Shang, J. 2017. Low-cost magnetic herbal biochar: characterization and application for antibiotic removal. *Environmental Science and Pollution Research*, **24**(7), 6679-6687.

Liu, S., Xu, W.-h., Liu, Y.-g., Tan, X.-f., Zeng, G.-m., Li, X., Liang, J., Zhou, Z., Yan, Z.-l., Cai, X.-x. 2017. Facile synthesis of Cu(II) impregnated biochar with enhanced adsorption activity for the removal of doxycycline hydrochloride from water. *Science of The Total Environment*, **592**, 546-553.

Mao, H., Wang, S., Lin, J.-Y., Wang, Z., Ren, J. 2016. Modification of a magnetic carbon composite for ciprofloxacin adsorption. *Journal of Environmental Sciences*, **49**, 179-188.

Rostamian, R., Behnejad, H. 2018. Insights into doxycycline adsorption onto graphene nanosheet: a combined quantum mechanics, thermodynamics, and kinetic study. *Environmental Science and Pollution Research*, **25**(3), 2528-2537.

Wu, S., Zhao, X., Li, Y., Zhao, C., Du, Q., Sun, J., Wang, Y., Peng, X., Xia, Y., Wang, Z., Xia, L. 2013. Adsorption of ciprofloxacin onto biocomposite fibers of graphene oxide/calcium alginate. *Chemical Engineering Journal*, **230**, 389-395.
